# A Review of Pediatric Lower Extremity Data for Pedestrian Numerical Modeling: Injury Epidemiology, Anatomy, Anthropometry, Structural, and Mechanical Properties

**DOI:** 10.1155/2018/6271898

**Published:** 2018-09-04

**Authors:** Yunzhu Meng, Costin D. Untaroiu

**Affiliations:** Department of Biomedical Engineering and Mechanics, Virginia Tech, Blacksburg, VA, USA

## Abstract

Pedestrian injuries are the fourth leading cause of unintentional injury-related death among children aged 1 to 19. The lower extremity represents the most frequently injured body region in car-to-pedestrian accidents. The goal of this study was to perform a systematic review of the data related to pedestrian lower extremity injuries, anatomy, anthropometry, structural, and mechanical properties, which can be used in the development of new pediatric computational models. The study began with a review of epidemiologic data related to pediatric pedestrian accidents. Anatomy of the child lower extremity and age-related anthropometry data were presented as well. Then, both the mechanical and structural properties of the lower extremity main components (e.g., bones, cartilages, knee ligaments, muscles, tendons, and growth plates) available in literature were summarized. The study concluded with a brief description of current child pedestrian models, which included a discussion about their limitations. We believe that data included in this review study can help in improving the biofidelity of current child models and support the development and validation of new child models used by safety researchers for protection of pediatric population.

## 1. Pedestrian Injury Facts

**Figure 1:**
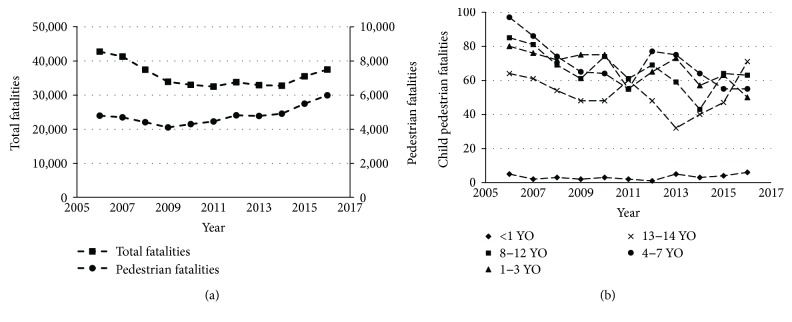
US traffic fatalities facts: (a) total traffic fatalities and pedestrian fatalities [2]; (b) child pedestrian fatalities [3].

About 1.25 million traffic deaths are recorded globally every year; 50% of which are vulnerable road users: pedestrians, cyclists, and motorcyclists [1]. While the number of US traffic fatalities per year decreased from 42,708 (2006) to 37,461 (2016), pedestrian fatalities remained almost unchanged and even increased recently from 4779 (2013) to 5987 (2016) (Figure 1(a)). In 2015, 4.3% of all pedestrian fatalities (233 of 5376) were children under 14 years old. Children in the age groups 0–4 and 5–9 years old were the highest percentage of those killed (21%) [2]. The latest child pedestrian traffic crash fatality data were based on traffic safety fact data collected in 2016 [3]. Children were classified into five age groups: less than 1, 1–3, 4–7, 8–12, and 13-14-year-olds, and each age group took about 2%, 20%, 23%, 26%, and 29% of child pedestrian fatality in traffic accidents, respectively (Figure 1(b)). Overall, pedestrian injury is the fourth leading cause of unintentional injury-related death for children aged 1 to 19 in the US [4].

Pedestrian accidents involving children between 4 and 12 years old occur most frequently in midblock areas or at intersections [5]. Injuries to child pedestrians are influenced by factors such as vehicle design, road environment, traffic engineering, child behavior [5], and temporal factors (e.g., time of day and season of year).

**Figure 2:**
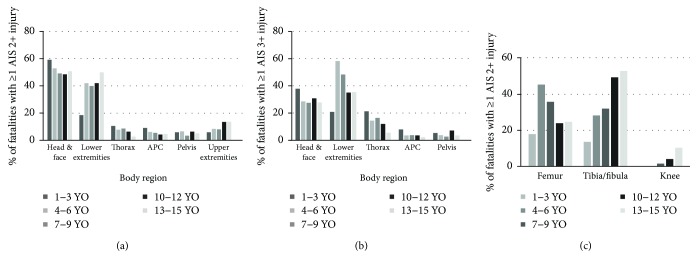
Percentage injury distribution in child pedestrian fatalities: (a) AIS 2+; (b) AIS 3+; (c) AIS 2+ in lower extremities [7, 8].

Pedestrians are usually impacted by the front end of the vehicle [6]. The head and lower extremity are the most likely regions to suffer moderate and serious injuries (AIS 2+) in child pedestrian accidents [7, 8] (Figure 2(a)). Serious injuries (AIS 3+) in lower extremities are frequently recorded in child pedestrians; the children in the age groups 4–6 and 7–9 years had the highest percentage (Figure 2(b)). Femur fractures and tibia/fibula fractures occur most frequently in the age range 4–6 years and 13–15 years, respectively (Figure 2(c)) [7].

While a child headform impact test is currently being used in pedestrian protection regulations [9], the rapid advancement in both computational power and nonlinear finite element (FE) technology could promote a computational component to supplement safety tests. Therefore, this review focuses on child data available in literature regarding mechanical properties that can be used in the development of child lower extremity models corresponding to both preschool (3- to 5-year-old) and middle-aged (6- to 12-year-old) children. When limited data or even lack of data for some lower limb structures were observed, test data collected on young animal or human adult samples were also reviewed. In addition, component test data that can be used in the validation of child lower extremity models were reviewed as well.

## 2. Lower Limb Anatomy and Injuries

### 2.1. Child Anthropometry Data

Pedestrian protection methods could differ based on child age. The anthropometry data included in this review paper are mostly based on three-, six-, and ten-year-old children. The popular growth charts (50th percentile data) for these three ages are summarized and compared with the corresponding data of the Hybrid III Anthropometric Test Device (ATD), which are currently used in regulatory crash tests (Table 1) [10–13].

Average lengths of the femur and tibia differentiated by gender were reported from a series of sixty-seven children [14]. The 50th percentile length data of the femur and tibia were also recorded on a series of 113 subjects (52 girls and 61 boys) [15]. Tibia and femur lengths of Korean children at the ages of 3 to 16 years were reported in literature as well [16] (Table 2). Both 50th percentile data, which is calculated as the middle point of a number set, and the average data reported in literature were summarized (Table 2).

### 2.2. Anatomic Differences between Child and Adult

Development of the human body occurs continuously from birth to adulthood. Body proportions change significantly during this growth period. For example, the child head is about 1/4 of the total body length at birth but only 1/7 in adult [17]. The midpoint of the body is at the umbilicus level in the newborn, but it moves below the umbilicus during the growth. Lower extremity length also increases with respect to the length of the torso and upper extremity during growth. Girls typically experience pubertal growth spurt between 11 and 13 years old becoming taller than boys with the same age [18]. However, similar body proportions were reported in children of either sex up to 10 or 11 years old [17].

Bones change from child to adult in terms of shape and size. A newborn's skeleton consists mostly of cartilage which ossifies into bone as the body matures. Initially, metaphyses of the long bones are separated by a thin layer called a growth plate, which is invisible in X-ray scans until it begins to ossify. The presence of a growth plate is considered a reference point to differentiate immature bone from mature bone [19]. In modeling, the growth plate requires careful attention because this cartilage layer is the weakest area of the young skeleton [20]. The thickness of the child femoral head growth plate decreases from 4 to 0.5 mm with age (Table 3) [21]. Lower variations in growth plate thickness (up to 12 years old) were reported for the fibula and tibia with average sizes of 3.1 mm and 3.9 mm, respectively (Table 4) [22].

**Figure 3:**
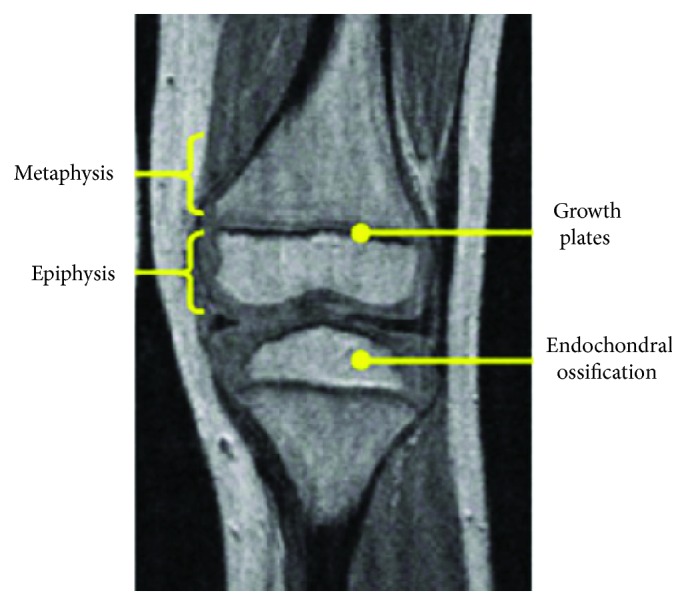
Child long bones MRI (figure based on [24]).

Although the data in Tables 3 and 4 were measured from X-ray images [23], MRI scans better capture growth plate width (Figure 3) [24]. Therefore, MRI scans of the lower extremity long bones are recommended for use in the development of numerical models.

**Figure 4:**
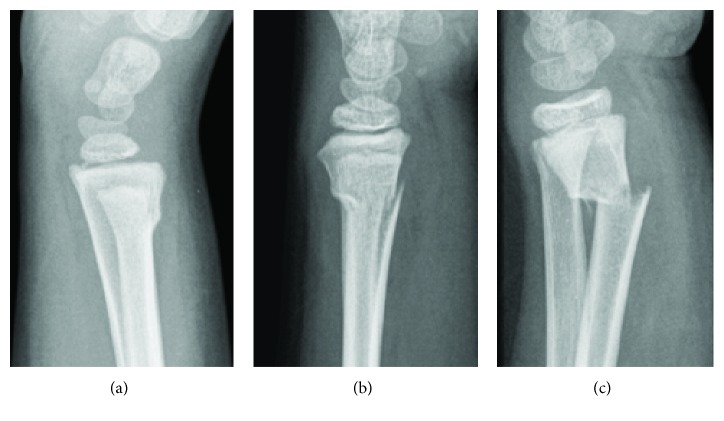
Three metaphyseal fracture patterns: (a) buckle; (b) greenstick; (c) complete fracture (figure based on [28]).

Pediatric long bones have a higher percent of trabecular bone and are surrounded by a thick periosteum. As a child grows up, ossification occurs, and some of the trabecular bone converts to cortical bone [25]. Higher ultimate strains were observed in child bone samples than in adult bone samples [26]. The periosteum makes the bone more plastic that affects the fracture pattern (Figure 4). Buckle fracture is an incomplete fracture type, which typically occurs near the metaphysis of the bone (Figure 4(a)). The side of the bone under compression crunches down upon itself causing the bone to crumple on one side [27, 28]. Child bones are more likely than adult bones to fracture in buckling due to higher elasticity. Greenstick fracture occurs when a bone bends and cracks, instead of breaking completely into separate pieces (Figure 4(b)), being more common in children than adults. The low ratio between the mature and the immature enzymatic cross-links in child bone tissue is a potential explanation for the higher presence of greenstick fractures in children [29]. Finally, a complete fracture could occur in the metaphysis region as well (Figure 4(c)).

## 3. Material Properties of Pediatric Lower Extremities

Material properties are usually derived based on load and displacement data recorded on specimens under simple loading (tension, compression, and shear). Optimization algorithms can be used to identify the most appropriate parameters for constitutive equations resulting in the best fit between computational and experimental stress-strain curves. The material properties of pediatric lower limb components (e.g., bone, cartilage, ligaments, muscles, tendons, and growth plates) are reviewed in the following sections.

### 3.1. Bone

The bone is an inhomogeneous, anisotropic, and viscoelastic material [30]. Incomplete ossification affects pediatric material properties making it elastic and rubbery [19]. Several tests were performed on child bone samples to identify the relationship between bone material properties (e.g., density, Young's and Poisson's moduli, etc.) and age. The cortical and trabecular bones have different mechanical characteristics, so tests were usually performed separately on samples of each bone type. However, due to limited pediatric data available in literature, tests performed on whole bone samples were included in this study as well. Compressive, bending, and tensile properties were summarized separately due to bone anisotropic properties.

#### 3.1.1. Bone Density

**Figure 5:**
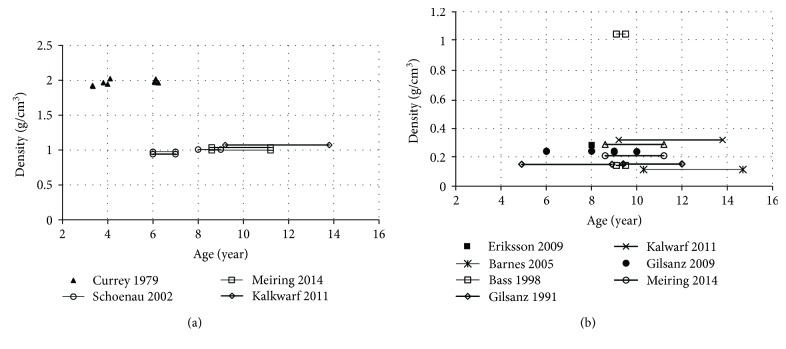
Density versus age: (a) cortical bone; (b) whole bone and trabecular bone (circle marks). Note: lines were used to show the average data for a certain age interval.

The bone density data obtained from either child volunteers [31–37] or post mortem human subject (PMHS) samples [38] were summarized in Figure 5. Bone mineral density (BMD) was measured in *in vivo* tests from healthy child volunteers using dual-energy X-ray absorptiometry (DXA) [31–33, 35, 36] or quantitative computed tomography [34, 37]. Then, the bone density was derived based on BMD by (1). The PMHS data were obtained from subjects who had been in bed less than 3 weeks before death, so cause of death is believed to not lead to changes in the bone [38].

(1) $$\begin{array}{c} Density=BMD\times \frac{4}{\left(\pi \times width\right)}. \end{array}$$

Although a slight increase in bone density was observed from childhood to early adulthood, no significant dependence with age [39, 40], gender [41], or race [34] among the children was shown.

#### 3.1.2. Compressive Material Properties of Bone

**Figure 6:**
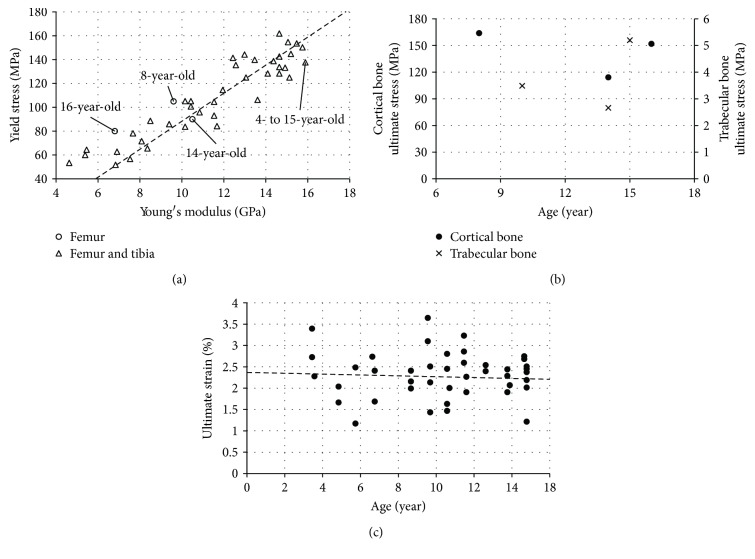
Long bone mechanical properties under compression tests: (a) yield stress versus Young's modulus for the femoral or tibial cortical bone [26, 42]; (b) ultimate stresses versus age [42, 43]; (c) ultimate strain versus age [26].

Compressive material properties of the pediatric cortical bone were reported by two studies [26, 42]. In the first study [26], cortical bone samples were collected from femoral and tibial shafts of 12 children (age range: 4 to 15 years, mean value 10.35 years) and 12 adult subjects (age range: 22 to 62 years). Cylindrical samples (3 mm diameter and 18 mm height) were compressed at a 0.1 s^−1^ strain rate until failure or until a 5% reduction of the specimen height was observed. The stress-strain curves were derived based on the time histories of load and extensometer displacement recorded during the testing. In children, the Young's modulus was reported as 12 GPa (34% lower than adult's data). The average values of yield stress and the ultimate stress showed to be lower for child bone than adult bone as 110 MPa (18% lower) and 130 MPa (33% lower). No significant difference was observed in the yield strain (1.1%) between pediatric and adult samples (Figure 6(a)), but the ultimate strain (*ε*_uc_, %) showed a decreasing trend with age (unit: year) (2) (Figure 6(c)). A relationship between the Young's modulus (*E*, unit: GPa) and yield stress (*σ*_y_, unit: MPa) was derived as (3) (Figure 6(a)).

(2) $$\begin{array}{c} \varepsilon_{uc}=2.37-0.01\times age. \end{array}$$

(3) $$\begin{array}{c} E=11.75\times \sigma_{y}+2.48. \end{array}$$

In addition, the Young's modulus, yield stress, ultimate stress (*σ*_uc_, unit: MPa), and yield strain (*ε*_yc_, %) were also correlated using polynomial relationships with respect to bone ash density (*ρ*, unit: mg/mm^3^) based on both child and adult subjects ((4), (5), (6) and (7)) [26]. While these data are very valuable for bone modeling, they have some limitations. Even though no visible lesions were observed in tested bone specimens, it should be mentioned that cancer was the cause of death of the child PMHS donors [26]. In addition, the results of 5 femur and 7 tibia pediatric tests were not reported separately, and the cylindrical specimens had lower dimensions (2 mm diameter and 14 mm height) in 4 tests due to the low thickness of the cortical wall.

(4) $$\begin{array}{c} E=12.9\rho^{2.0}, \end{array}$$

(5) $$\begin{array}{c} \sigma_{y}=125.2\rho^{2.2}, \end{array}$$

(6) $$\begin{array}{c} \sigma_{uc}=144.7\rho^{2.0}, \end{array}$$

(7) $$\begin{array}{c} \varepsilon_{yc}=1.05+0.08\rho . \end{array}$$

Compression tests were performed on the children long bone samples to investigate how a genetic disorder (osteogenesis imperfecta) affects the bone material properties [42]. Cortical bone samples collected from three healthy children PMHS (8-, 14-, and 16-year-old) were cut into parallelepipeds (approximately 3 mm long, 1.6 mm wide, and 1.6 mm high) and used as a control group. The samples were initially loaded to 100 N, then unloaded and reloaded until fracture at a 0.7 *μ*m/s displacement rate. The Young's modulus, defined as the slope of linear unloading portion, was derived from the three bone samples of the 8-year-old child PMHS as 9.6 GPa. The corresponding average yield and ultimate stresses were reported as 105 MPa and 163.9 MPa (Figures 6(a) and 6(b)), respectively.

Tests were also performed to investigate the age effect on the material properties of the trabecular bone [43]. Samples of lumbar and calcaneus trabecular bone were collected from 150 PMHS between 10 and 90 years old. Cubical calcaneus samples (1.27 cm size) from a 10-year-old boy and three other PMHS children between 10 to 15 years old were included in this study. Dependence between the ultimate stress and the age was observed (Figure 6(b)).

Due to the limited number of pediatric PMHS, surgical waste was employed to investigate the cortical bone mechanical behavior. 15 fibula and 7 femur samples from 21 children (1–18 years old, mean 9.7 ± 5.8 years old) were employed in the tests [44]; all the samples were fairly small (2 × 2 × 2 mm^3^), so regular mechanical tests cannot be performed. Ultrasonic method then become an alternative; compressive elastic coefficients were calculated based on the wave velocity measured and were summarized in Table 5.

#### 3.1.3. Bending Material Properties of Bone

Bending tests were performed on cortical bone samples of 9 PMHS under 14 years old [45]. Rectangular parallelepiped samples with approximately 23 to 26 mm length, 3 mm width, and 2 mm height were collected from the midfemoral shaft and then loaded under periosteal-endosteal direction at a 5 mm/min constant rate. The values of the Young's modulus for 4-, 6-, and 8-year-old femoral cortical bone were reported as 98.5, 137.8, and 122.7 GPa. High values of bone Young's modulus in this study could be caused by neglected shear effect on relatively low span/depth values of tested specimens [46]. The ultimate stresses were recorded as 176.8, 207.2, and 190.4 MPa, respectively. Both bending strength and Young's modulus increased steadily with age (up to 90 years old), except the specimens from a six-year-old child who suffered from diabetes, and its bone properties may be affected.

Young's modulus, bending strength, and failure moment were derived based on bone density [47]. Four specimens (4 mm) were collected from the left femur shafts. The average wet densities (*ρ*_wet_) were measured as 1.64, 1.76, 1.84, and 1.82 g/cm^2^ corresponding to pediatric subjects of 2.5, 13, 15, and 16 years old, respectively. Based on an empirical Young's modulus-density relationship derived from the tibial bone [48], the Young's modulus (*E*) was calculated as 10.2, 13.4, 15.5, and 15.5 GPa. Finally, for the same pediatric data, the ultimate stress was calculated as 212, 233, 247, and 244 MPa based on another empirical relationship of ultimate stress versus elastic modulus [48, 49].

Three-point bending tests were performed on coupons collected from 9 PMHS (age: 3–16 years old) [50]. Transversely isotropic properties of bone were considered, so bone specimens were tested along both longitudinal and circumferential directions. Five cycles of preconditioning at 0.2 mm/min were performed for each test, and a 2 mm/min ramp was used to failure. Typical load-displacement curves were published, and the average Young's modulus, yield strength, and ultimate strength were derived as 4.4 ± 0.4 GPa, 61.4 ± 5.3 MPa, and 83.0 ± 7.8 MPa along the longitudinal direction and 1.6 ± 0.4 GPa, 20.8 ± 6.0 MPa, and 26.5 ± 8.6 MPa along the circumferential orientation. Finally, it should be mentioned that all tested PMHS used in this study suffered bone disease (osteogenesis imperfecta) which may result in bone fragility [50].

**Figure 7:**
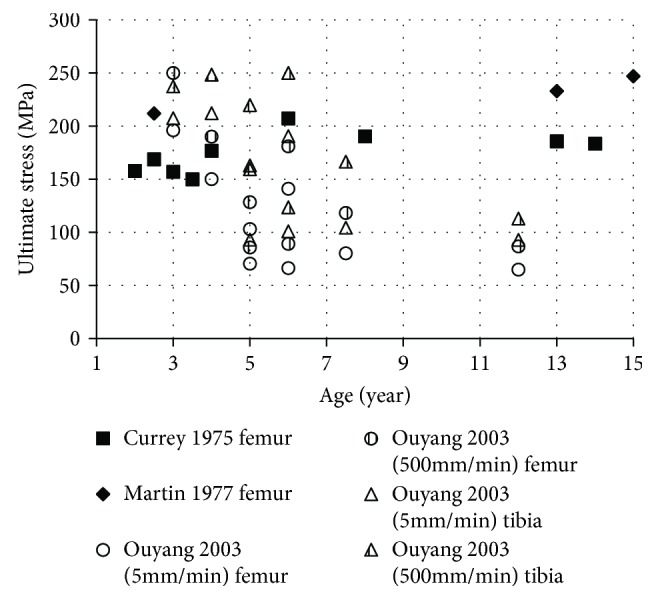
Ultimate stress versus age (open circle: estimate values) (∆: tibial data; all other marks: femoral data) [45, 47, 51].

The failure force of bone was also reported from the three-point bending tests of femur and tibia specimens collected from 11 children aged 2.5 to 12 years [51]. To compare this data with ultimate stress data published in literature, the bending stress was estimated based on the average length of the femur/tibia reported in literature [16] (Figure 7).

Three-point micro-bending tests were performed on cortical bone samples extracted from 7 children fibulas (age 4–16 years old) [52, 53] collected from surgery waste. All 18 rectangular samples had dimensions in the following ranges: 15–35 mm length, 10–20 mm width, and 2-3.5 mm thickness. The average Young's modulus of all samples was reported as 15.5 ± 3.4 GPa in the transverse direction (*E*_t_) and 9.1 ± 3.5 GPa in the longitudinal direction (*E*_l_). Additionally, a linear relationship between these parameters derived by curve-fitting (*R*^2^ = 0.72) as

(8) $$\begin{array}{c} E_{t}=0.84\times E_{l}+8.14. \end{array}$$

The Poisson's ratio was reported as 0.24 ± 0.08 in transverse direction.

Additional three-point micro-bending tests were further performed by the same team on 18 fibular cortical bone samples collected from 8 children patients (age: 5–16 years old, mean 10.6 ± 4.4 years) [54]. The ranges of sample dimensions were 13–32 mm in length, 4–10 mm in width, and 1–3 mm in thickness. The average static modulus of elasticity assessed from three-point micro-bending tests was reported as 9.1 ± 3.5 GPa. The dynamic modulus of elasticity and Poisson's ratio derived based on ultrasonic protocol were reported as 15.5 ± 3.4 GPa and 0.24 ± 0.08, respectively.

#### 3.1.4. Tensile Material Properties of Bone

Fifteen dumbbell-shaped samples with 2.5 mm width, 0.5 mm thickness, and a total 25.4 mm length were harvested from a 15-year-old PMHS femur and tibia [55]. The samples were loaded in tension quasi-statically (0.01 mm/s) and dynamically (15 mm/s) along the longitudinal direction. The engineering strain and stress were derived based on the time histories of sample deformation and tensile force. The average values of bone stiffness and failure parameters are reported relative to sample location (e.g., lateral femur, medial femur, and tibia) (Table 6) [55].

### 3.2. Articular Cartilage

#### 3.2.1. Tensile Properties of Articular Cartilage

**Figure 8:**
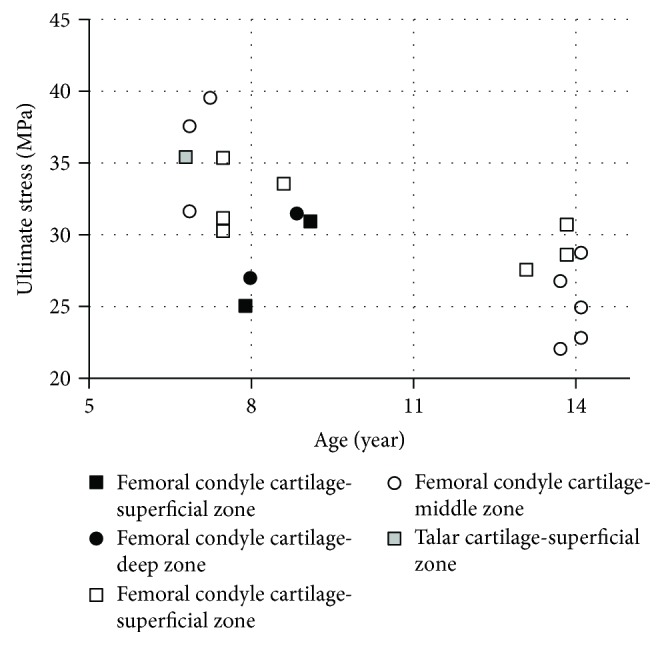
Articular cartilage tensile ultimate stress versus age: □: superficial zone; ○: middle and deep zones [56, 57].

**Figure 9:**
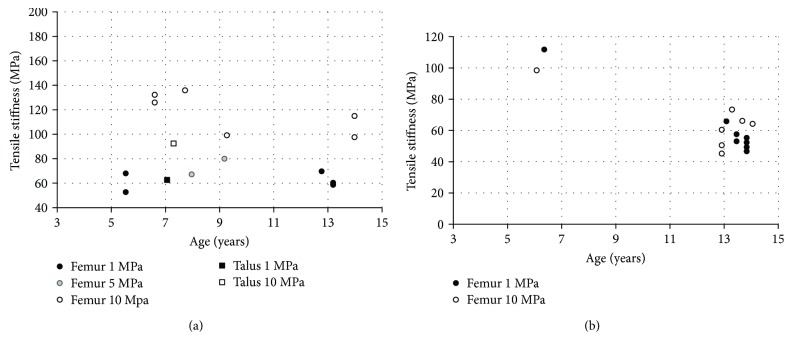
Articular cartilage: tensile stiffness versus age under 1, 5, and 10 MPa loading stress: (a) superficial zone; (b) middle zone [56, 57].

Articular cartilage is a white smooth tissue that covers the ends of long bones in the joint region. Its function is to facilitate the load transmission in joint with very low friction and wear. The mechanical and material properties of articular cartilage are depth dependent, so it is usually investigated separately based on superficial zone and deeper zone. Based on our knowledge, only two studies have reported pediatric material properties of articular cartilage in the knee [56, 57] and ankle [57]. In the first study [56], tensile data were reported from testing specimens of articular cartilage obtained from the femoral condyle regions of PMHS aged 8–91 years. A total of 24 knee joints were cut into the shape of a dumbbell (10 mm length by 200 *μ*m thickness in total; gauge has a 0.5 mm width and a 3 mm length). The dumbbell specimens were loaded at 5 mm/min constant rate along the predominant alignment of the collagen fibers in the superficial zone. In the second study [57], similar tests were performed on specimens of articular cartilage removed from the femoral head and the talus of PMHS aged 7–90 years. Tensile stiffness was derived as the gradient to the stress versus strain curve. Overall, both tensile stiffness and ultimate stress show a decreasing trend with age. The data derived from pediatric specimens show higher values than the corresponding adult properties, especially in the femoral head (Figures 8 and 9) (Table 7).

#### 3.2.2. Compressive Properties of Articular Cartilage

Based on our knowledge, pediatric material properties under compressive loads have not been reported in literature. However, several tests based on animal samples were performed to investigate immature articular cartilage material properties [58, 59]. A series of compressive tests were performed on 143 immature rabbit samples (0–3 months) [58]. Articular cartilage samples were collected from the tibial medial plateaus and femoral medial condyles. The samples were loaded under 0.01 N for 30 minutes and then were compressed in four steps with a step-load of 0.04 N. The compressive properties of both the tibial and femoral cartilage showed age-related dependency with an increase from 0.2 to 0.6 MPa during growth (newborn to 3 months).

Compressive tests were also performed on immature bovine (1-2 weeks) articular cartilage samples [59] collected from both superficial zone and deeper zone of the medial and lateral condyles and from the patellofemoral grooves. All the samples were cut into cylinders with 3 mm diameter. The thickness of superficial zone and deeper zone samples were 200–400 *μ*m and 1300 *μ*m, respectively. Preinjury mechanical properties were measured by three compressive tests with final strains of 10%, 12.5%, and 15%. Then, samples were compressed to an injurious final strain (50%) at 100%/sec. The equilibrium modulus and dynamic stiffness of the superficial zone samples were recorded to be lower than those of the deeper zone samples before injury occurred. During injurious compressive tests, the ultimate stress of the superficial zone samples (0.88 MPa) was obviously lower than that of the deeper zone samples (13.5 MPa). Meanwhile, the superficial zone samples compressed more (20.31%) than deeper zone samples (7.8%) during injury.

### 3.3. Growth Plate

In pediatric long bones, the growth plate is a thin hyaline cartilage that separates the shaft from the epiphyses and distributes the load placed on osseous units. Growth plates have different mechanical properties than the bone [60–62]. Therefore, distinct growth plate models should be incorporated into pediatric long bone models to better predict long bone injuries. The material properties of growth plate were reported in literature [63]. The data measured from child PMHS samples were rare [64, 65], so the research using animal specimens were also included to emphasize the anisotropic material properties of growth plate. All the animal specimens were collected from mammals such as rats [64, 66], rabbits [67], pigs [68, 69], and calves [61, 62].

#### 3.3.1. Tensile Properties

Growth plate samples extracted from the femoral heads of two male human subjects (8- and 14-year-old) were tested under tensile loading along the axis of the femoral head [65]. Quasi-static tests were performed with the load rates as 0.0004, 0.004, and 0.04 mm/s. Eight of the forty samples prepared for the tests failed through the cartilage. One sample was from an 8-year-old subject, and the other seven were from a 14-year-old subject. Tangent modulus, defined as the slope of the steepest portion of the stress-strain curves, ultimate stress, and ultimate strain were derived from the test data (Table 8). It should be mentioned that both subjects suffered cerebral palsy which may affect the material properties.

**Figure 10:**
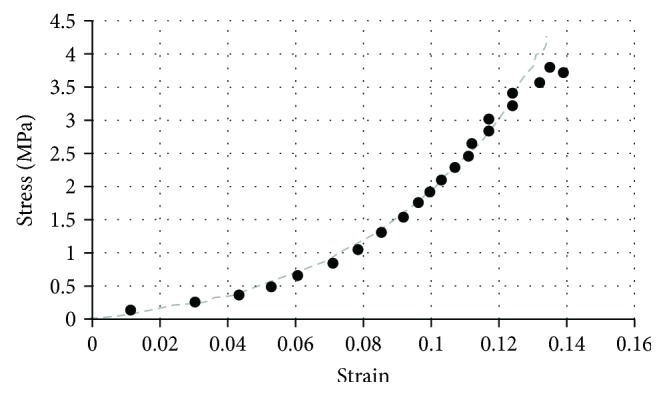
A typical tensile stress-strain curve of a bone growth plate bovine specimen [61].

Due to the limited number of human samples available, several tests using bovine specimens were summarized as follows. The human and bovine growth plates have anatomic similarities in structure and similar overall shape of stress-strain curves [70]. The tensile stress-strain relationship of bovine femoral growth plate tested along the growth direction showed an exponential shape: *σ* = *A*(*e*^*Bϵ*^ − 1), where *σ* is the engineering stress and the *ε* is the Green-Lagrangian strain (Figure 10) [61]. All specimens (7 mm × 7 mm) were obtained from twelve distal femora of immature bovine (~12 months old). Two tangent moduli from 86 tensile tests (~0.004 s^−1^ strain rate) were reported: the toe-region tangent modulus (at 0% strain) and the tangent modulus at 75% of the ultimate strain (Table 9).

Lower tensile strength, stiffness, and ultimate strain were observed in the growth plate than in the articular cartilage and meniscus (Table 9). In addition, tensile material properties of the growth plate appear to be region-specific (Table 9).

Among the tests performed at different strain rates on proximal tibia of 4 bovine (12 to 18 months), the lateral region had a greater tangent modulus than the medial and central regions. In addition to the anatomical location, the tensile material properties were shown to vary by strain rate and age (Table 10). No significant difference was observed in ultimate stress among specimens [65]. During the growth process, the growth plate gets thinner; tangent modulus decreases; and the ultimate strain and stress get higher (Table 11). A relationship between ultimate stress (*σ*) and growth plate thickness was proposed as follows:

(9) $$\begin{array}{c} \sigma \left(MPa\right)=3.2-2.8\times growth\;plate\;thickness\;\left(mm\right). \end{array}$$

#### 3.3.2. Compressive Properties

To the best of author's knowledge, no compressive tests were performed on human growth plate specimens. Several tests performed on animal specimens (calf and swine) were summarized as follows to provide basic material property information of the growth plate.

Twelve specimens were collected from 5-month-old calf proximal tibial growth plates [71]. The specimens were first preloaded to 1 N, then unconfined compression at 0.055 mm/min was applied until 20% strain and held 1400 sec to record a complete stress relaxation cycle. The mechanical behavior of growth plate was modeled as articular cartilage by biphasic theory [72] as a mixture of an elastic isotropic solid and an inviscid fluid. The Young's modulus (*E*) of the isotropic material was identified by an inverse finite element analysis as 1.1079 ± 0.3990 MPa [73] (Table 12). A relatively close value of Young's modulus (1.08 MPa) was identified from other stress-relaxation compression tests performed on calf samples [62]. The values of permeability coefficient (*k*) and Poisson ratio (*ν*) (Table 12) were reported as well.

A transversally isotropic material model for the solid matrix of growth plate with the plane of transverse isotropy perpendicular to the loading direction showed a better fit to the test data [62]. The out-of-plane Young's modulus (*E*_3_) was derived directly based on the data of an unconfined test; it was assumed *ν*_31_ = 0, and then a three-parameter optimization procedure was used to extract the transverse permeability coefficient (*k*_1_), the Young's modulus in the transverse plane in tension (*E*_1_), and the Poisson's ratio (*ν*_21_) (Table 13).

Similar stress-relaxation tests in unconfined compression were performed on porcine distal ulnae growth plates [69]. The cylindrical samples were initially preloaded at 5% strain then a 15% strain at a strain rate 1.5 × 10^−3^s^−1^. A similar transversely isotropic biphasic model was assumed, and its parameters were obtained directly from test data (*E*_3_) or by analytical model (*E*_1_, *k*_1_, *ν*_21__,_*ν*_31_) (Table 13).

#### 3.3.3. Bending Properties

**Figure 11:**
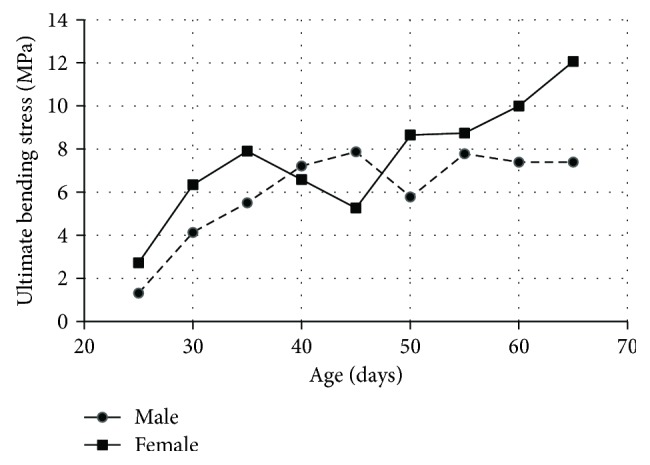
Rat tibia growth plate: ultimate bending stress versus age [60].

To the best of author's knowledge, the bending properties of human growth plate are lacking in literature, so animal (rat) data were summarized in this section. Bending tests were performed on the proximal ends of rat tibias (280 rats, 560 tibiae) using a variable strain-rate machine (20 mm/s). The time histories of load and displacement were recorded during testing, and ultimate bending stress was derived from the general formula for bending stress of an elastic beam *σ* = *Mc*/*I* (M: maximum bending moment, I: the moment of inertia, and c: the distance from the neutral axis to the outermost fiber). It was observed that the ultimate bending stress tends to increase with age, and the female growth plate was usually stronger in bending than the male growth plate (Figure 11) [60].

Similar bending tests were performed on 380 rats [74]. A tangential force was applied on the upper femur epiphysis until the epiphysis separated from the shaft. As in previous tests on rat tibias [60], the maximum bending stresses increased with age. The average mechanical strength grows from 70.8, 87.2, to 135.3 g/mm^2^ for the samples of 10-, 12-, and 15-week-old rats.

#### 3.3.4. Shear Properties

**Figure 12:**
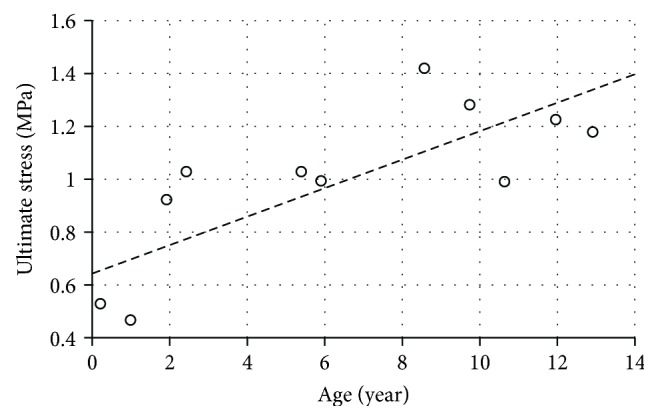
Ultimate stress versus age under shearing tests using human femoral head specimens [64].

Shearing tests were performed on pediatric femoral heads to determine the shear strength of the growth plate [64]. The femoral proximal ends were collected from 25 child PMHS aged five days to 15 years. Load was applied in an anterior-posterior direction over the secondary center of ossification of the femoral head until failure occurred. One sample from each pair was tested after removing the perichondrial fibrocartilaginous complex as the excised group; the other one was tested with the complete fibrocartilaginous complex as the control group. Failure loads were compared between two groups, and it showed that the existence of perichondrial fibrocartilaginous complex makes the femur head stronger in shearing. For the samples collected from a six-year-old child, the failure loads were recorded as 696.5 N for the control specimen and 539.6 N for the excised specimen. Then, the ultimate stress for the control specimen was derived as 0.988 MPa based on the measured cross-sectional area (7.04 cm^2^). Based on the control group data, a linear regression relationship between the ultimate shear stress (*τ*) and age was derived (Figure 12)

(10) $$\begin{array}{c} \tau \left(MPa\right)=0.656+0.055\times Age\;\left(Year\right). \end{array}$$

Due to the limited number of human specimens, the shearing data obtained on porcine growth plate specimens were reviewed as well. Shearing tests were performed (0.05 mm/sec) on nine 5-month-old porcine proximal femoral head specimens with variable loading directions (lateral, vertical, and anterior) [75]. Force and deformation were recorded until failure (Table 14). No cross-section area data were measured in each test, so the anterior ultimate stress was calculated based on an estimated cross-section area of 961.6 mm^2^.

### 3.4. Tendons

Tendons are tough bands of fibrous connective tissues that connect muscles to bones and serve to stabilize joints (e.g., knee joint). Similar to ligaments, tendons are made of collagen and can withstand tensile loads. Overall, tendons are usually modeled as either nonlinear or linear materials. The stiffness and Young's modulus of a tendon increases during childhood and influences muscular force transfer to the skeleton. Tendon injuries (ruptures) usually appear in tension [76], so their tensile properties are critical for the tendon numerical models.

#### 3.4.1. Achilles Tendon

Since the Achilles tendon is one of the most frequently injured tendons in the human body, its tensile material properties were investigated using isolated coupons or *in vivo*. The stress versus strain curves obtained from tensile tests in failure on Achilles tendons collected from a 13-year-old and a 15-year-old PMHS were reported [77]. The Young's moduli, calculated as the slopes of the reported stress versus strain curves, were about 1800 MPa for the 13-year-old child specimen and 645 MPa for the 15-year-old child specimen. The ultimate stress and strain were recorded as 50.90 MPa and 3.39% for the 13-year-old child specimen, and 44.23 MPa and 6.85% for the 15-year-old child specimen.

In an *in vivo* study [78], 53 volunteer children between the ages of 5 and 12 years were seated on a dynamometer chair, and the ankle moment was measured using an isokinetic dynamometer. Then, the tensile load was calculated as the ratio of the ankle moment and the moment arm estimated using the tendon excursion method. The tendon elongation was measured as the displacement of the gastrocnemius medialis muscle-tendon junction (GM MTJ) using ultrasound images. The tendon stiffness and elastic modulus, calculated based on the slope of the line fitted to the force-elongation data between 10% and 90% of peak force, have the following equations:

(11) $$\begin{array}{c} stiffness\;\left(N/mm\right)=18.172\times age\;\left(Years\right)-10.514, \end{array}$$

(12) $$\begin{array}{c} E\left(MPa\right)=64.303\times age\;\left(Years\right)-1.553. \end{array}$$

Calculated by (11) and (12), the tensile stiffness for 3-, 6-, and 10-year-old PMHS tendons are estimated as 44.0, 98.5, and 171.2 N/mm, and the Young's moduli were calculated as 191.4, 384.3, and 641.5 MPa, respectively. An alternative way to determine stiffness was also reported [78]. Data between 10% and 90% of peak force of the second weakest participant (corresponding to a force range of 54 to 484 N) were used to calculate the absolute stiffness, but lower correlation was observed (*R*^2^ = 0.15 lower than the relation reported in (12) *R*^2^ = 0.37).

Similar tests were performed to understand how resistance training influence the properties of the child Achilles tendon [79]. Similar to the test procedure shown above [78], tendon properties of ten children (8.9 ± 0.3 years of age) were measured and used as control group data. All the mechanical properties were measured twice, at both beginning and end of the test duration (10 weeks). The mean values of the stiffness were recorded as 162.5 and 167.4 N/mm, and the Young's modulus was 629.4 and 663.1 MPa.

Tensile tests were performed on tendon specimens collected from all ages [80]. The pediatric specimens were groups according to ages (0–9-year-old and 10–19-year-old). Ultimate strength and ultimate elongation were recorded as 51.98 MPa and 11% for the younger group and 54.92 MPa and 10% for the elder group.

#### 3.4.2. Patellar Tendon

Material properties of the patellar tendon were also identified *in vivo* on 21 elementary children (average: 11.2-year-old), 18 junior high school students (average: 13.8-year-old) and 22 adults [81]. Both stiffness and Young's modulus, calculated based on the forces and elongations measured in tests, showed an increase during growth. Their mean values were 742.9 N/mm and 533.6 MPa for the elementary children group and were 1211.9 N/mm and 867.4 MPa for the junior high school students group.

**Figure 13:**
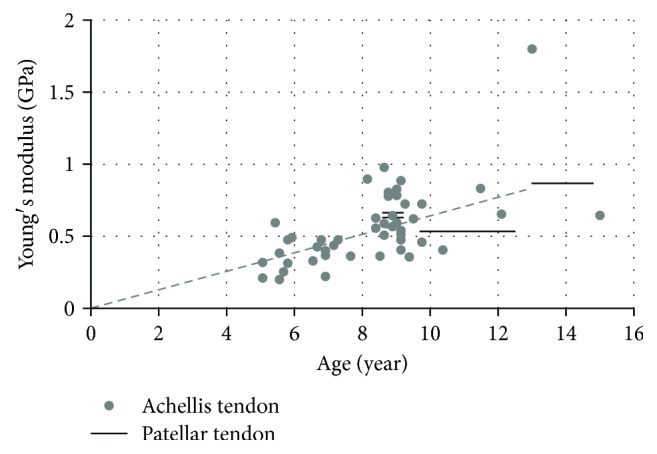
Young's modulus of tendon versus age [77–79, 82]. (Note: lines were used to show the data for a certain age interval).

The elastic properties of human tendons were measured *in vivo* on nine younger boys (average: 10.8-year-old), nine elder boys (average: 14.8-year-old), and 14 young adults [82]. The Young's modulus of patellar tendon were compared with the Achilles tendon properties [77–79], and a steady increase was shown during growth (Figure 13).

#### 3.4.3. Other Tendons

Tensile tests to failure were also performed on toe and finger coupons collected from both children and adults (43 individuals in total) [83]. Compared to the adult material properties, the pediatric specimens showed lower tensile strength (29–44 MPa) and a greater ultimate strain (14–18%). The Young's modulus of pediatric specimens (0.343 GPa) was three times lower than in adult tendons.

### 3.5. Knee Ligaments

No test data regarding material properties of pediatric human knee ligaments were identified in literature. A review of adult human knee ligament properties is briefly summarized in this section. However, the reader is referred to Weiss and Gardner [84] for a more detailed review.

Ligaments are anisotropic material with higher stiffness along the collagen fibers. Other than one test reported in literature [85], the ligaments were tested along the longitudinal direction (the direction of collagen fibers). Tensile tests corresponding to two age groups (6 specimens from young adult at the ages of 16–26 years; 20 specimens from old adult at the ages of 48–86 years) were performed on the anterior cruciate ligament (ACL) until failure at strain rate of 1.0 s^−1^ [86]. A decrease in terms of tangent modulus, ultimate strain, and stiffness relative to donors' ages was observed (Table 15). The material properties of the two posterior cruciate ligament (PCL) bundles (aPC: anterolateral bundle; pPC: posteromedial bundle) were determined separately [87]. Specimens collected from adult PMHS (53–98-year-old) were tested in tension until failure, and their material properties were derived (Table 15). Similar tensile tests were performed on the human lateral collateral ligament (LCL), PCL, ACL, and medial collateral ligament (MCL) specimens (Table 15) [85, 88].

### 3.6. Skeletal Muscles

Skeletal muscles are soft tissue that have the ability to contract and cause movement. Most of the skeletal muscles are attached to the bone. Material properties of the pediatric lower limb muscles (mostly stiffness) were reported only from different *in vivo* tests. For example, ultrasound shear wave electrography (SWE) was used to measure muscle stiffness *in vivo* [89]. Each child of thirteen healthy participants (age range: 2–11.5-year-old, median age: 5.3-year-old) was assisted in a prone position. Transducer was placed with low pressure on the calf skin to ensure the skeletal muscle was in a relaxation phase. Then, the foot was rotated, and the measurements were performed when the target positions (20°, 10°, and 0° plantar flexion) were reached. Finally, shear Young's modulus was reported (Table 16) for the median, first, and third interquartile range (Q1 and Q3).

A combination of ultrasonography and motion analysis was also used to derive mechanical properties of the pediatric plantar-flexor muscles *in vivo* [90]. Each child of ten participants (age 12.0 ± 2.9 years) was assisted in a seated position on a dynamometer chair with the right knee fully extended. The right ankle joint was rotated by the dynamometer system with constant angular velocities as 1, 10, and 30°/s. Muscle elongation was measured using ultrasonography, and the stiffness was then investigated (Table 17).

## 4. Structural Properties of Pediatric Lower Extremities

The structural properties are usually reported as force-displacement curves from tests of entire components (e.g., tibia, femur) or the whole human body. These properties vary based on factors such as specimen anthropometry and cannot be assigned directly to FE models. However, this valuable data could be used to calibrate or validate the models of lower limb components or the whole lower limb model. Therefore, the pediatric structural properties of lower limb components identified in literature were reviewed in the following sections.

### 4.1. Long Bone under Bending Loading

**Figure 14:**
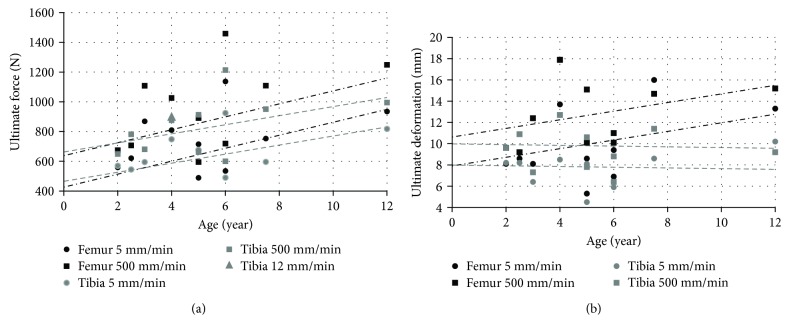
Ultimate force and deformation under three-point bending femur and tibia tests: (a) ultimate force; (b) ultimate deformation [51].

Three-point bending tests were performed on lower extremities long bones extracted from 11 PMHS aged 2 to 12 years old [51] and included two 3-year-old children and two 6-year-old children. Both ends of the long bones (femur and tibia) were potted in cups. Each long bone was loaded at the midshaft location until fracture by an impactor driven by a universe machine (SWD-10) at constant speeds (5 mm/min or 500 mm/min). The impact force and impactor displacement at the time of bone fracture were reported for each tested bone (Figure 14).

Three-point bending tests were also performed on PMHS thighs [91]. The majority of PMHS subjects were under 1 year old, but thighs corresponding to a 3-year-old PMHS and a 6-year-old PMHS were tested as well. Each thigh was loaded quasi-statically with a constant velocity of 50 mm/min at the midthigh location until fracture. Ultimate forces were recorded as 1744 and 2016 N for right and left thighs of the 3-year-old PMHS, and their corresponding deformations were 38 mm. For the left thigh from the 6-year-old PMHS, the ultimate force was recorded as 2920 N, and the ultimate deformation was recorded as 60 mm.

Intact tibiae and those with drill-holes extracted from a 4-year-old PMHS and four 14-year-old PMHS were also tested in three-point bending tests with a 12 mm/min impactor speed [92]. The ultimate force for the 4-year-old intact tibia was reported as 902 N. The ultimate forces of 14-year-old intact tibiae were recorded as 7649, 5492, 7502, and 6816 N. Overall, ultimate force and deformation increase almost linearly during growth under bending loads.

### 4.2. Pelvis under Lateral Impact

**Figure 15:**
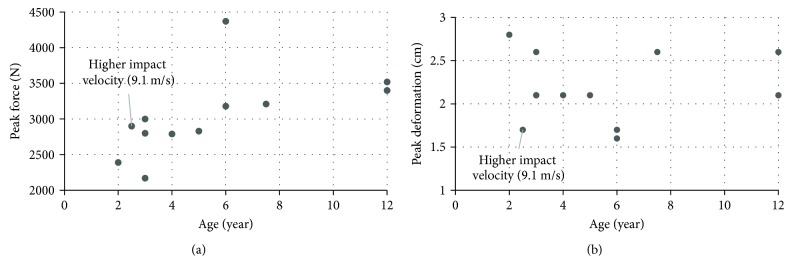
Peak force and deformation under lateral impact: (a) peak force; (b) peak deformation [93] (Note: two 3-year-old PMHS have the peak deformation as 2.1 cm which coincide in figure (b)).

Pelvic behavior under lateral impact was investigated in a test series which included PMHS from 2 years old up to 12 years old (specifically, three PMHS were at the age 3 years and two PMHS were 6 years old) [93]. The PMHS were set in a seated posture and then were loaded laterally by a rectangular impactor (18 cm wide by 14 cm high). The impact speeds were set between 7 and 8 m/s during all tests except one which had a higher impact velocity (9.1 m/s). Ultimate force (Figure 15(a)), ultimate deformation (Figure 15(b)), and corresponding force-deformation curve were reported for each specimen. An increasing trend of peak force versus age was observed.

## 5. Numerical Child Pedestrian Models

To develop improved technologies for child pedestrian protection, a subsystem impact test with child headform impactor was introduced in regulations in Europe and Asia [9]. In addition, test targets which mimic a running child (5 km/h) are used in a new test for evaluation of pedestrian automatic emergency braking (AEB) systems introduced in 2016. While an adult pedestrian dummy (50th male Polar ATD) was designed and evaluated against PMHS tests [94–96], no child pedestrian ATD has been developed yet. Rapid advancement in computational power makes numerical child pedestrian models important tools for safety research. Two types of child pedestrian models were reported in literature: multibody (MB) models and finite element (FE) models.

**Figure 16:**
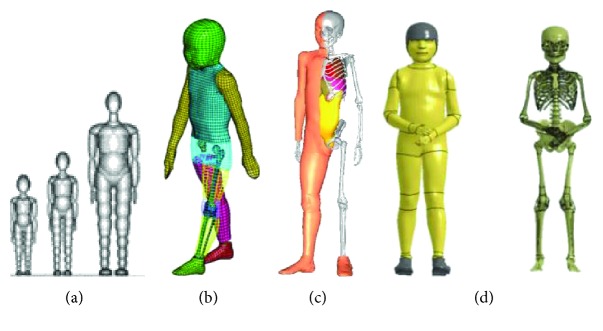
Numerical child pedestrian models: (a) 3- and 6-year-old child MB models compared to a midsize male model (left to right) (figure based on [97]); (b) six-year-old child FE lower extremity model (figure based on [24]); (c) CHARM-10 child pedestrian FE model [103]; (d) GHBMC 6YO-PS child pedestrian FE model (figure based on [104]).

MB systems approximate the human body as a set of rigid bodies with predefined inertial properties connected by various types of joints in an open-loop system known as a “tree structure”. A software package called the MADYMO (TASS, Rijswijk, The Netherlands) was used to develop all the MB models reviewed below. The development of several child MB models for 3-, 6-, 7-, 9-, and 15-year-old child pedestrians were reported in literature [97–99] (Figure 16(a)). These relatively low computational cost models were used to improve the accident reconstruction of child pedestrians [100]. The injuries in these models could be predicted only at the predefined locations using locked joints with failure defined based on maximum loads.

Currently, FE models are the most sophisticated human numerical models. In addition to better geometry and contact force representation, these models can model the deformation of their components and predict the local distribution of stress and strain. A number of pedestrian models have been developed in various FE software, either covering the complete body or focusing on the lower limbs.

In 2003, Okamoto et al. developed a FE model for the 6-year-old child pedestrian using the PAM-CRASH (ESI Group, Paris, France) (Figure 16(b)) [24]. The lower extremity models were developed in detail based on MRI scans obtained from a child volunteer. Specific child anatomical features were implemented in the model (e.g., the ossification centers at the femoral head, femoral and tibial condyles, ankle, the epiphyseal cartilage at ends of the femur and tibia, and growth plates). The menisci geometries were obtained from the MRI scans, and an anatomy book was referenced as the geometry source of the knee ligament models (ACL, PCL, LCL, and MCL). The pedestrian model was validated based on a pedestrian accident reconstruction study [101].

THUMS (Total Human Model for Safety) Version 4 models, released in 2010, included a 6-year-old child pedestrian FE model. All THUMS models were developed with the LS-DYNA software (LSTC, Livermore, CA, USA). However, little information about the development and validation of THUMS child pedestrian model was provided in literature [102].

CHARM-10 occupant and pedestrian models corresponding to a 10-year-old child were recently released (Figure 16(c)) [103]. Anthropometry data and internal measurements of bony structures were based on clinical images (CT and MRI). The model has a stature of 140 cm and weight of 35 kg. The FE model includes 993 parts, 949,311 nodes, and 1,678,610 elements. Material properties were based on literature resources or scaled from adult data. Validation was performed at component level (e.g., pelvis, pelvic girdle, long bones, lower legs, and knee in the lower limbs). The kinematic response (head impact time) showed reasonable agreement with a MADYMO model in a car-to-pedestrian (CPC) simulation, but no quantitative validation was performed.

A 6-year-old pedestrian FE model (GHBMC 6YO-PS) was also recently released (Figure 16(d)) [104, 105]. The geometry of the child model is based on literature sources [106] and surface scan data [107] of a 6-year-old child (117 cm height and 23.86 kg weight). The model includes more than 580 parts, 538,000 nodes, and 834,000 elements. Material properties of long bones' cortical bone were assigned based on literature sources [45, 108]. Validations were performed on component level (e.g., pelvis, long bone, and knee) and then CPC were performed under various impact velocities (20–60 km/h). The head impact time and wrap around distance were compared among the simulations and showed reasonable results. In addition, it showed that assigning adult material properties [109–112] to a pediatric lower extremity model results in stiffer prediction than PMHS data.

## 6. Discussion and Conclusion

Children differ structurally from adults in several ways that are critical to address before studying pediatric pedestrian protection. To support the development of accurate numerical pedestrian models, child anthropometry data and skeletal differences were summarized in this review. The body of knowledge of material properties of bones, cartilages, knee ligaments, skeletal muscles, tendons, and growth plates in pediatric lower extremities was collated. Published experiments were investigated; static and dynamic properties were sorted according to the tissue type. Due to the lack of child PMHS data corresponding to the growth plate, knee ligament, and cartilage specimens, animal tests were investigated instead. Structural properties based on component tests were also included, aimed at calibrating and validating the child models. Based on reviewed experimental data, pediatric tissue properties are usually different from adult tissue. Therefore, pediatric material properties obtained from testing or scaling adult data should be used while modeling the pediatric lower extremities. Current child pedestrian models were summarized and discussed in this paper as well. We believe that the reviewed data may help to improve the biofidelity of current pediatric models and support the development and validation of new pediatric models. However, the reader should be also informed about the reduced number of tests for many pediatric tissues which may influence the predictions of pedestrian model. Thus, more tests using child PMHS are strongly recommended to be performed in the future to support the validation of child models. To investigate and improve the safety protection for the whole child population, numerical models for various ages and gender should be developed. Therefore, in addition to listing the recorded material properties, regression equations relative to age are encouraged to be developed and then used in these future age-dependent child models.

## Figures and Tables

**Table 1:** Child anthropometric data: stature (cm)/weight (kg).

	3 YO	6 YO	10 YO	Reference
Boy	96.1/14.3	116.0/20.5	137.8/31.2	[10, 11]
Girl	95.1/13.9	115.1/20.2	138.6/31.9	[10, 11]
Boy	98.8/15.9	119.5/23.1	142.5/38.0	[12]
Girl	99.0/15.4	118.8/22.5	144.5/39.0	[12]
Hybrid III dummy	95.3/16.2	116.8/23.4	137.4/35.2	[13]

**Table 2:** The average/50th percentile length of children lower extremity bones reported in literature (cm) (boy/girl).

	3 YO	6 YO	10 YO	Reference
Femur^∗^	21.09/21.29	28.09/28.52	36.29/36.72	[14]
Femur^∗^	22.31/22.85	30.09/29.34	36.77/36.52	[16]
Femur^∗∗^	19.84	27.14	35.20	[15]
Tibia^∗^	16.79/16.81	22.12/22.53	28.53/29.28	[14]
Tibia^∗^	17.95/17.87	23.35/22.53	28.77/28.66	[16]
Tibia^∗∗^	16.29	22.11	29.21	[15]
Fibula^∗∗^	16.18	21.93	28.68	[15]

Note: ∗: average length; ∗∗: 50th percentile length.

**Table 3:** Growth plate thickness versus age (femoral head) [21].

Age (years)	Femoral head growth plate thickness (mm)
0–4	1–4
5–9	1
10–14	0.5–1
15–19	0.5

**Table 4:** Growth plate width versus age (fibula and tibia) [22].

Age (years)	Fibula growth plate width (mm)	Tibia growth plate width (mm)
0–3	3.0 ± 0.6	4.1 ± 0.4
3–6	2.9 ± 0.7	3.9 ± 0.8
6–9	3.5 ± 0.9	4.2 ± 1.0
9–12	3.2 ± 1.1	3.3 ± 0.8
Mean	3.1 ± 0.8	3.9 ± 0.9

**Table 5:** Mechanical properties of femoral and fibula cortical bone under bending.

	*C*_11_ (GPa)	*C*_22_ (GPa)	*C*_33_ (GPa)	*C*_44_ (GPa)	*C*_55_ (GPa)	*C*_66_ (GPa)
Femur	12.2 ± 2.42	12.9 ± 3.15	19.0 ± 5.50	3.57 ± 0.833	3.31 ± 0.921	2.77 ± 0.656
Fibula	16.5 ± 2.70	15.8 ± 3.24	24.0 ± 5.15	4.17 ± 0.800	4.05 ± 0.746	3.13 ± 0.373

**Table 6:** Tensile mechanical properties of the femur/tibia [55].

Specimen region and number of samples		Young's modulus (GPa)	Yield strain (%)	Failure strain (%)	Yield stress (MPa)	Failure stress (MPa)	Strain rate (%/s)
Femur lateral	5	15.98 ± 0.94	0.88 ± 0.08	1.68 ± 0.23	139.64 ± 8.41	161.66 ± 7.54	0.06 ± 0.001
Femur medial	4	14.53 ± 2.65	0.90 ± 0.16	2.20 ± 0.96	128.33 ± 3.01	147.05 ± 10.80	0.08 ± 0.01
Tibia lateral	2	17.50 ± 0.28	0.91^∗^	1.40 ± 1.00	178.2^∗^	148.4 ± 38.89	0.05 ± 0.02
Tibia medial	2	15.75 ± 0.49	0.97 ± 0.03	1.48 ± 0.35	153.40 ± 0.99	164.85 ± 0.21	0.04 ± 0.002
Tibia posterior	2	14.35 ± 0.35	0.89 ± 0.06	1.33 ± 0.29	127.15 ± 4.88	144.30 ± 1.84	0.06 ± 0.004

^∗^Data collected from tests performed on a 15-year-old PMHS samples.

**Table 7:** Tensile mechanical properties of the pediatric articular cartilage.

Age (years)	Region	Superficial zone		Middle and deep zones		Reference
		Ultimate stress (MPa)	Tensile stiffness at 10 MPa applied stress (MPa)	Ultimate stress (MPa)	Tensile stiffness at 10 MPa applied stress (MPa)	
7	Femoral condyle	33	150	32^∗^	60–100^∗^	[57]
8	Femoral condyle	25	136	27		[56]
9	Femoral condyle	31	98	32		[56]
8	Talar cartilage	24	125	17.5^∗^		[57]

∗: Middle zone mechanical properties.

**Table 8:** Tensile material properties of the human capital femoral growth plate [65].

Growth plate thickness (mm)	Ultimate stress (MPa)	Ultimate strain (%)	Tangent modulus (MPa)
1.35 ± 0.33	0.98 ± 0.29	31 ± 7	4.26 ± 1.22

**Table 9:** Tensile material properties of the growth plate and comparison with the articular cartilage and meniscus (bovine specimens) [61].

Region	Toe tangent modulus (MPa)	Tangent modulus at 75% of ultimate strain (MPa)	Ultimate stress (MPa)	Ultimate strain
Anterior	20.6 ± 14.7	48.6 ± 25.1	4.10 ± 0.97	0.137 ± 0.05
Posterior/lateral	16.9 ± 9.6	37.9 ± 16.7	3.05 ± 0.80	0.123 ± 0.04
Posterior medial	9.9 ± 6.2	23.5 ± 14.9	2.3 ± 0.68	0.160 ± 0.06
Center	18.6 ± 11.2	27.0 ± 11.8	2.16 ± 0.79	0.117 ± 0.06
*Growth plate average*	**15.5 ± 3.2**	**34.6 ± 17.7**	**2.97 ± 0.80**	**0.138 ± 0.06**
*Articular cartilage*	**—**	**84.0**	**25.0**	**>0.4**
*Meniscus*	**—**	**200.0**	**>30.0**	**>0.1**

**Table 10:** Tensile material properties of the growth plate by regions (bovine tibia specimens) [65].

Strain rate	Region	Tangent modulus	Ultimate stress (MPa)
0.04 mm/s	Lateral	15.78 ± 1.66	3.46 ± 0.45
	Center	11.81 ± 2.12	2.64 ± 0.29
	Medial	12.63 ± 2.79	2.60 ± 0.92

0.004 mm/s	Lateral	14.60 ± 2.22	2.82 ± 0.42
	Center	9.53 ± 2.38	2.01 ± 0.50
	Medial	11.75 ± 3.71	2.18 ± 0.64

0.0004 mm/s	Lateral	13.22 ± 3.74	2.56 ± 0.35
	Center	9.81 ± 2.16	2.05 ± 0.43
	Medial	11.10 ± 2.36	2.25 ± 0.60

**Table 11:** Tensile material properties of the growth plate by age (bovine specimens) [65].

Age (months)	Growth plate thickness	Tangent modulus	Ultimate strain (%)	Ultimate stress (MPa)
5	0.65 ± 0.12	7.52 ± 2.15	23 ± 6	1.36 ± 0.53
12–18	0.50 ± 0.15	6.89 ± 1.96	38 ± 13	1.82 ± 0.55

**Table 12:** Growth plate: compressive material properties using isotropic material type.

	*E* (MPa)	*ν*	*k* (×10^−15^m^4^/Ns)	Reference
Calf	1.08	0	15.5	[62]
	1.1079 ± 0.3990	—	—	[72]

**Table 13:** Growth plate: compressive material properties using transversely isotropic biphasic material type.

	*E*_3_ (MPa) compression	*E*_1_ (MPa) tension	*ν*_21_	*ν*_31_	*k*_1_ (×10^−15^m^4^/Ns)	Reference
Swine	0.51 ± 0.12	8.65 ± 1.72	0.24 ± 0.07	0.08 ± 0.03	1.82 ± 0.67	[69]
Calf	0.47 ± 0.11	4.55 ± 1.21	0.30 ± 0.20	0.0	5.0 ± 1.8	[62]

**Table 14:** Porcine femoral head specimens: ultimate load, ultimate deformation, and estimated ultimate stress under variable loading direction [75].

Load direction	Ultimate load (N)	Ultimate deformation (mm)	Estimated ultimate stress (MPa)
Anterior	1756	7.4	1.83
	1750	7.5	1.82
	1679	8.7	1.75
Mean (SD)	**1728 ± 43**	**7.9**	**1.80**
Lateral	1148	7.1	
	918	7.0	
	920	7.1	
Mean (SD)	**995 ± 132**	**7.1**	
Vertical	7397	5.0	
	7413	6.6	
	7008	6.4	
Mean (SD)	**7273 ± 229**	**6.0**	

**Table 15:** Material properties of the adult human knee ligaments.

Ligament	Age range/average age (years)	Tangent modulus (MPa)	Tensile ultimate stress (MPa)	Tensile ultimate strain (%)	Reference
ACL	48–86	65.3 ± 24.0	13.3 ± 5.0	48.5 ± 11.9	[86]
	16–26	111 ± 26	37.8 ± 9.3	60.25 ± 6.78	
	27	312.58	34.73	14.97	[88]
PCL					
aPC	75	75	35.9 ± 15.2	18.0 ± 5.3	[87]
pPC	27	248 ± 119	24.4 ± 10.0	19.5 ± 5.4	
	27	145 ± 69	37.94	15.86	[88]
LCL	27	362.12	35.75	13.35	[88]
MCL	62	332.2 ± 58.3	38.6 ± 4.8	17.1 ± 1.5	[85]
	62^∗^	11.0 ± 3.6	1.7 ± 0.5	11.7 ± 0.9	

^∗^Load under transverse direction.

**Table 16:** Shear Young's modulus of child skeletal muscles [89].

	Q1 (kPa)	Median (kPa)	Q3 (kPa)
Age (years)	4.3	5.3	9.4
Position			
20° plantar flexion	6.1	7.8	11
10° plantar flexion	7.3	9.6	15.6
0° plantar flexion	10.9	14.9	20.9

**Table 17:** Mechanical properties of child skeletal muscles [90].

Age (years)	Resting muscle length (cm)	Muscle stiffness (N/cm)
12.0 ± 2.9	17.9 ± 3.5	38.1 ± 21.4
